# Impact of COVID-19 pandemic on Tuberculosis and HIV services in Ghana: An interrupted time series analysis

**DOI:** 10.1371/journal.pone.0291808

**Published:** 2023-09-20

**Authors:** Eric Osei, Hubert Amu, Gideon Kye-Duodu, Mavis Pearl Kwabla, Evans Danso, Fred N. Binka, So Yoon Kim

**Affiliations:** 1 Department of Population and Behavioural Sciences, Fred N. Binka School of Public Health, University of Health and Allied Sciences, Ho, Ghana; 2 Department of Epidemiology and Biostatistics, Fred N. Binka School of Public Health, University of Health and Allied Sciences, Ho, Ghana; 3 Department of Policy Planning Monitoring and Evaluation, Mental Health Authority, Accra, Ghana; 4 Asian Institute for Bioethics and Health Law, College of Medicine, Yonsei University, Seoul, Korea; University of Gondar College of Medicine and Health Sciences, ETHIOPIA

## Abstract

**Introduction:**

The Coronavirus disease 2019 (COVID-19) burden, coupled with unprecedented control measures including physical distancing, travel bans, and lockdowns of cities, implemented to stop the spread of the virus, have undoubtedly far-reaching aftereffects on other diseases. In low and middle-income countries (LMICs), a particular worry is the potential impact on Human Immunodeficiency Virus (HIV) and Tuberculosis (TB), as a consequence of possible disruption to health services and limiting access to needed life-saving health care. In Ghana, there is a paucity of information regarding the impact of COVID-19 on disease control, particularly TB and HIV control. This study sought to contribute to bridging this knowledge gap.

**Method:**

The study involved the analysis of secondary data obtained from the District Health Information Management System-2 (DHIMS-2) database of Ghana Health Service, from 2016 to 2020. Data were analysed using an interrupted time-series regression approach to estimate the impact of COVID-19 on TB case notification, HIV testing, and Antiretroviral Therapy (ART) initiations, using March 2020 as the event period.

**Results:**

The study showed that during the COVID-19 pandemic period, there was an abrupt decline of 20.5% (955CI: 16.0%, 24.5%) in TB case notifications in April and 32.7% (95%CI: 28.8%, 39.1%) in May 2020, with a median monthly decline of 21.4% from April-December 2020. A cumulative loss of 2,128 (20%; 95%CI: 13.3%, 26.7%) TB cases was observed nationwide as of December 2020. There was also a 40.3% decrease in people presenting for HIV tests in the first month of COVID-19 (April 2020) and a cumulative loss of 262620 (26.5%) HIV tests as of December 2020 attributable to the COVID-19 pandemic. ART initiations increased by 39.2% in the first month and thereafter decreased by an average of 10% per month from May to September 2020. Cumulatively, 443 (1.9%) more of the people living with HIV initiated ART during the pandemic period, however, this was not statistically significant.

**Conclusion:**

This study demonstrated that the COVID-19 pandemic negatively impacted TB case notifications and HIV testing and counselling services, However, ART initiation was generally not impacted during the first year of the pandemic. Proactive approaches aimed at actively finding the thousands of individuals with TB who were missed in 2020 and increasing HIV testing and counselling and subsequent treatment initiations should be prioritised.

## Background

The emergence of the severe Acute Respiratory Syndrome Coronavirus-2 (SARS CoV-2) and related response measures have indeed had an immense impact on the performance of healthcare systems globally [1]. Healthcare facilities are being overloaded, and there are growing demands for more medical supplies owing to the large increases in hospitalizations due to COVID-19 [2]. The implementation of unprecedented measures such as stringent restrictions on human mobility, social distancing, and quarantine, to ameliorate the situation, has resulted in an unintended reduction in access and utilization of health care services as well as disrupting disease control activities for many non-communicable diseases [1, 3].

The immediate impact of COVID-19 on communicable diseases, such as TB and HIV and AIDS is yet to be fully known. However, according to the WHO, the global TB epidemic is likely to worsen due to the intense pressures COVID-19 exerts on health systems, leading to the weakening of the national TB control programmes and the possible effect of the interaction of the two infections [4]. The WHO has suggested that the pandemic has led to a decline of at least 25% in TB case detection globally over 3 months, and TB mortality is expected to rise by about 13% from the 2015 levels in 2020, a significant drawback in the advance towards the End TB strategy goals and targets [5]. The evidence available from several high TB burden countries suggests huge declines in the monthly TB cases notified in 2020, especially in Indonesia, India, Sierra Leone, and South Africa due to COVID-19 [5]. According to the WHO’s Global TB Report 2021, there was about a 1.3 million drop in the number of newly diagnosed TB cases and notified in 2020, an 18% decline from the previous year’s figure [6].

Further, it has been reported that access to routine HIV testing in many countries has declined due to COVID-19-associated response measures and this may negatively impact the implementation of the United Nations Programme on AIDS (UNAIDS) target of having 90% of all people living with HIV knowing their status [7].

Many scholars have estimated the impact of the COVID-19 pandemic on TB and HIV control in different settings. However, almost all of these studies employed descriptive approaches that are not robust and cannot directly infer causal effects [8–13].

In Ghana, many scholars have evaluated the impact of the COVID-19 pandemic on sectors such as education [14–16], business and economy [17–20], and Agriculture [21]. However, there is scanty information about the impact of COVID-19 on disease control programmes, particularly TB and HIV control in the country. This study, therefore, aimed to contribute to evidence generation to bridge this knowledge gap.

## Materials and methods

### Study design and setting

This study utilised an interrupted time-series analysis of routine national surveillance data in Ghana. Located in the West of the African continent, just north of the Equator, Ghana is divided into sixteen administrative regions and 265 districts with a projected population of 31, 732,129.

The diagnosis and treatment of TB and HIV/AIDS in Ghana are the responsibility of the National Tuberculosis Control Programme (NTP) and National AIDS Control Programme (NACP), respectively. Currently, TB control is integrated into the Ghana Health Service (GHS) structure at all levels of health care where TB diagnostic and treatment services are provided free of charge. Each region, district, or health facility has a team of health workers responsible for implementing TB control activities at that level. There is also a TB focal person at each of the levels who supervises the daily activities of the TB control programme.

HIV testing and counselling are institutionalised in public health facilities in Ghana, and the approaches used currently include provider-initiated testing and counselling, client-initiated testing and counselling, routine antenatal testing, community-based testing and counselling, assisted partner notification as well as other index case-based testing [22, 23], which are all provided free of charge. HIV testing is done according to national and international guidelines. HIV test results are recorded and those diagnosed as HIV-positive are referred for immediate ART services [23]. In this study, the interruption or intervention is the occurrence of the COVID-19 pandemic.

### Data source

Service data of TB patients and people tested and treated for HIV were extracted from the District Health Information Management System (DHIMS-2) database of Ghana Health Services. The DHIMS2 was rolled out in Ghana as a comprehensive Health Management Information System solution for routine data collection and compilation at all levels of health care to support decision-making. The platform, developed and implemented by the GHS in collaboration with the University of Oslo, is an internet-based open-source application that can be accessed by users with permission from the health facility. The database, which uses the tenet of a data warehouse and a modular structure that can be customized to the distinctive needs of different health systems, is used to remotely capture and compile routine clinical and public health data including morbidities, admissions, and mortalities health data across different levels of a health system [24]. Anonymised health data are entered at the health facility level only in customized user-defined forms based on a paper form and are aggregated into the facility, sub-district, district, regional, and national level summaries. The database has the added functionality of carrying out data quality analysis. Data entered are routinely validated by the District Health Management Team (DHMT) by running validation rules to identify violations. Where violations are detected, the health facility involved is notified to make corrections accordingly. TB and HIV and AIDS control programmes are considered special and have parallel reporting systems where health facilities designated to diagnose and manage them are mandated to report all activities to the district level monthly, which are entered into the DHIMS II database by district staff.

TB service data contained in the database include the number of TB cases notified during the month, type and category of TB, age grouping, sex, HIV status of TB patients, anti-retroviral treatment data and treatment outcomes. Similarly, HIV data captured in the database include the number of tested and counselled for HIV, age grouping, sex, HIV test result, cluster of differentiation 4 (CD4) cell count of HIV-positive persons, and anti-retroviral therapy initiations.

### Outcome measures

The primary outcome for TB control was the number of new and relapse TB cases notified per month while that for HIV control was the number of HIV tests per month and the number of people living with HIV who initiated Anti-retroviral therapy (ART) per month.

### Data handling and statistical analysis

National aggregated data were extracted directly from the database into Microsoft Excel 2010 spreadsheet for cleaning. Cleaning was done by visually inspecting the month-by-month data and checking for consistency and completeness of the data set before exporting it into STATA IC version 16 for analysis. Descriptive statistics were used to summarize demographic and clinical data and present crude summaries of outcomes before and after the pandemic. An interrupted time series analysis using an advanced mixed-effect linear regression model with robust standard errors that account for autocorrelation and heteroskedasticity [25] was used to assess COVID-19 effect on (1) TB case notification, (2) HIV Testing and counselling, and (3) ART initiation. Month-fixed effects regression was used to account for these variations or seasonal patterns of TB transmission that may occur within each month of a year. Additionally, month-fixed effects accounted for the predictable variations that are related to each month, leading to more reliable predictions and more accurate estimates of other model parameters.

The model was fitted for data from January 2016 to March 2020 as pre-pandemic and from April to December as the pandemic period. The model can be written as follows:

Where: that represents the random variability not explained by the model at a j^th^ time

**β**_**0**_: Baseline level (intercept) of the outcome variable at the beginning of the series (t = 0)

**β**_**1*i***_: "Slop" indicating the change in the trend of the outcome per time (Month) in the pre-COVID-19 segment (baseline trend)

**β**_**2*i***_: Represents the level change following the intervention

The fitted model was then used to predict the counterfactuals or expected cases from April 2020 to December 2020 in the absence of COVID-19. A 95% confidence interval of the counterfactuals was also estimated using estimated standard error from the fitted model at each time point and the standardized z-score deviate at a 95% confidence level. The absolute and relative differences between the counterfactual and the observed cases and the corresponding 95% confidence interval of the difference were also estimated. The model was used to predict the size of the month-by-month impact of the COVID-19 pandemic and the overall effect of the pandemic by the end of the year 2020. Separate models were built for each outcome of interest. March 2020 was used as the event (intervention) month since it was the month when COVID-19 cases were first reported in Ghana, and control measures were initiated.

### Ethical issues

The study was approved by the Yonsei University Health Systems, Severance Hospital Institutional Review Board (ID: 4-2021-1355) and the Ghana Health Service Ethical Review Committee (ID: GHS-ERC: 012/12/21) before data collection. Informed consent was not required in this study as there was no direct contact with participants. Permission to extract and use surveillance data was obtained from the Centre for Health Information Management unit of the Ghana Health Service. TB and HIV service data in the DHIMS2 database are aggregated and do not contain individual identifiers, hence it was impossible to identify patients.

## Results

### Impact of COVID-19 pandemic on TB case notification

**Fig 1:**
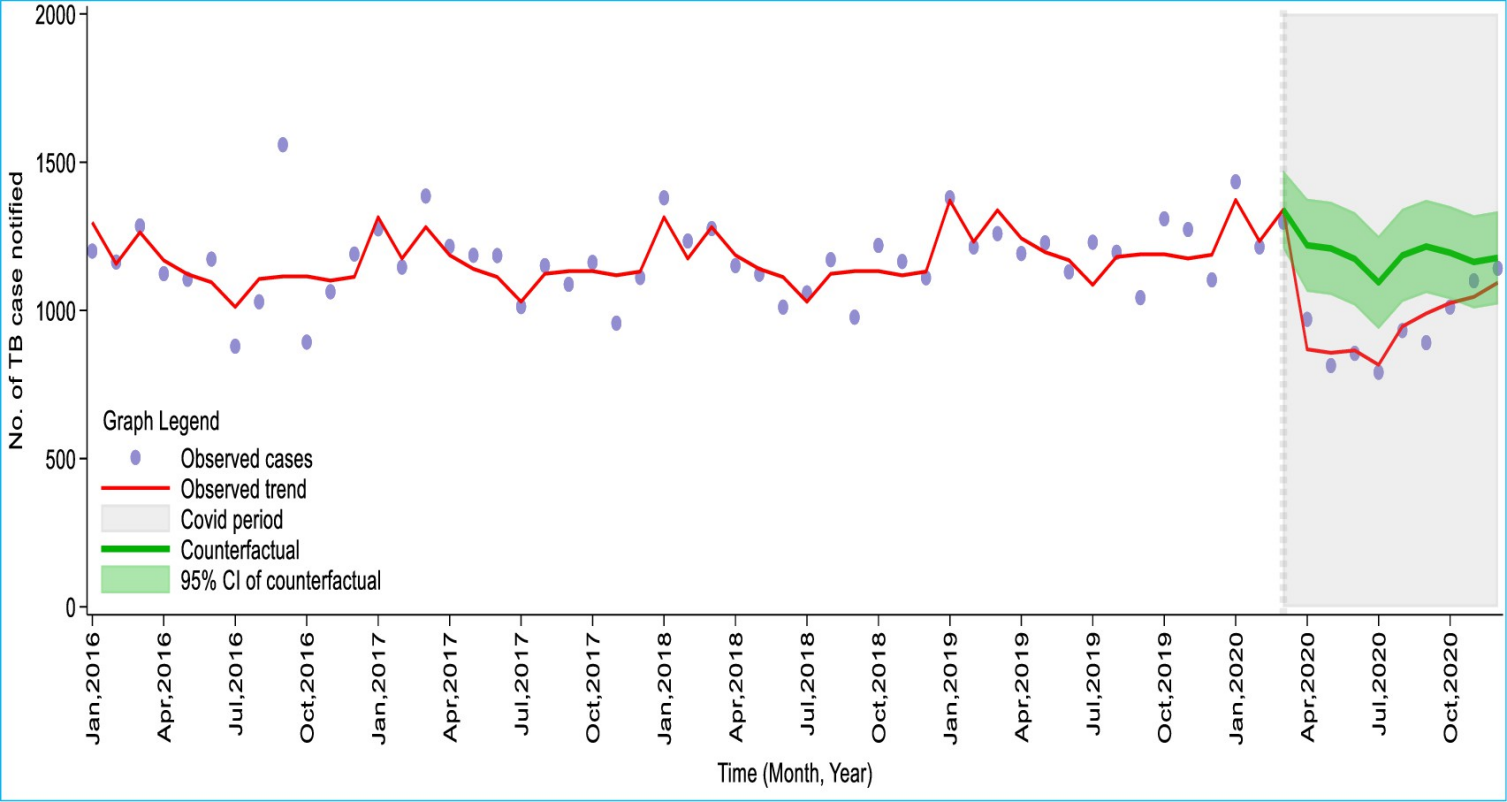
Trend of observed and counterfactual number of TB case notifications from January 2016 to December 2020 in Ghana.

In the 39 months before COVID-19 (January 2016 to March 2020), an average of 1,163 cases of TB were notified per month (Inter Quarter Range [IQR]: 680), compared with 932 cases per month (IQR: 351) in the 9 months of COVID-19 (April to December 2020). Table 1 and Fig 1 show the results of the modelled effect of the COVID-19 pandemic on TB cases notified. In the first month of COVID-19 (April 2020), 970 cases of TB were notified in Ghana. Under the counterfactual situation of no COVID-19 pandemic, 1,220 [95%CI: 1154, 1,285] TB cases would have been notified. The result, therefore, showed that COVID-19 led to an abrupt decline of 20.5% in TB case notifications in April, which further decreased to 32.7% the following month. Overall, a median of 21.4% (95%CI: 16.4%, 30.6%) of TB cases was lost or had delayed diagnosis per month during the COVID-19 period. A cumulative loss of 2,128 or 20% (15.3%, 24.2%) TB case notifications was observed nationwide as of December 2020 due to the COVID-19 pandemic.

**Table 1:** Modelled effect of COVID-19 pandemic TB case notification in Ghana, from April to December 2020.

	Observed No. of TB cases notified (Pandemic)	Counterfactual of No. of TB cases notified (No pandemic) [95% CI]	Difference	
Month			Absolute [95% CI]	Relative (%) [95% CI]
April	970	1220 [1154, 1285]	250 [184, 315]	20.5 [16.0, 24.5]
May	814	1209 [1144, 1274]	395 [330, 460]	32.7 [28.8, 36.1]
June	855	1174 [1108, 1239]	319 [253, 384]	27.2 [22.9, 31.0]
July	791	1094 [1029, 1159]	303 [238, 368]	27.7 [23.1, 31.8]
August	932	1186 [1120, 1251]	254 [188, 319]	21.4 [16.8, 25.5]
September	891	1216 [1150, 1281]	325 [259, 390]	26.7 [22.5, 30.4]
October	1011	1195 [1129, 1260]	184 [118, 249]	15.4 [10.5, 19.8]
November	1100	1163 [1098, 1229]	63 [-2, 129]	5.4 [-0.2, 10.5]
December	1142	1177 [1112, 1243]	35 [-30, 101]	3.0 [-2.7, 8.1]
Total	8,506	10634 [10044, 11221]	2,128 [1538, 2715]	20.0 [15.3, 24.2]
Median	932	1186 [1029, 1342]	254 [188, 319]	21.4 [16.4, 30.6]

### Impact of COVID-19 on HIV testing and counselling

**Fig 2:**
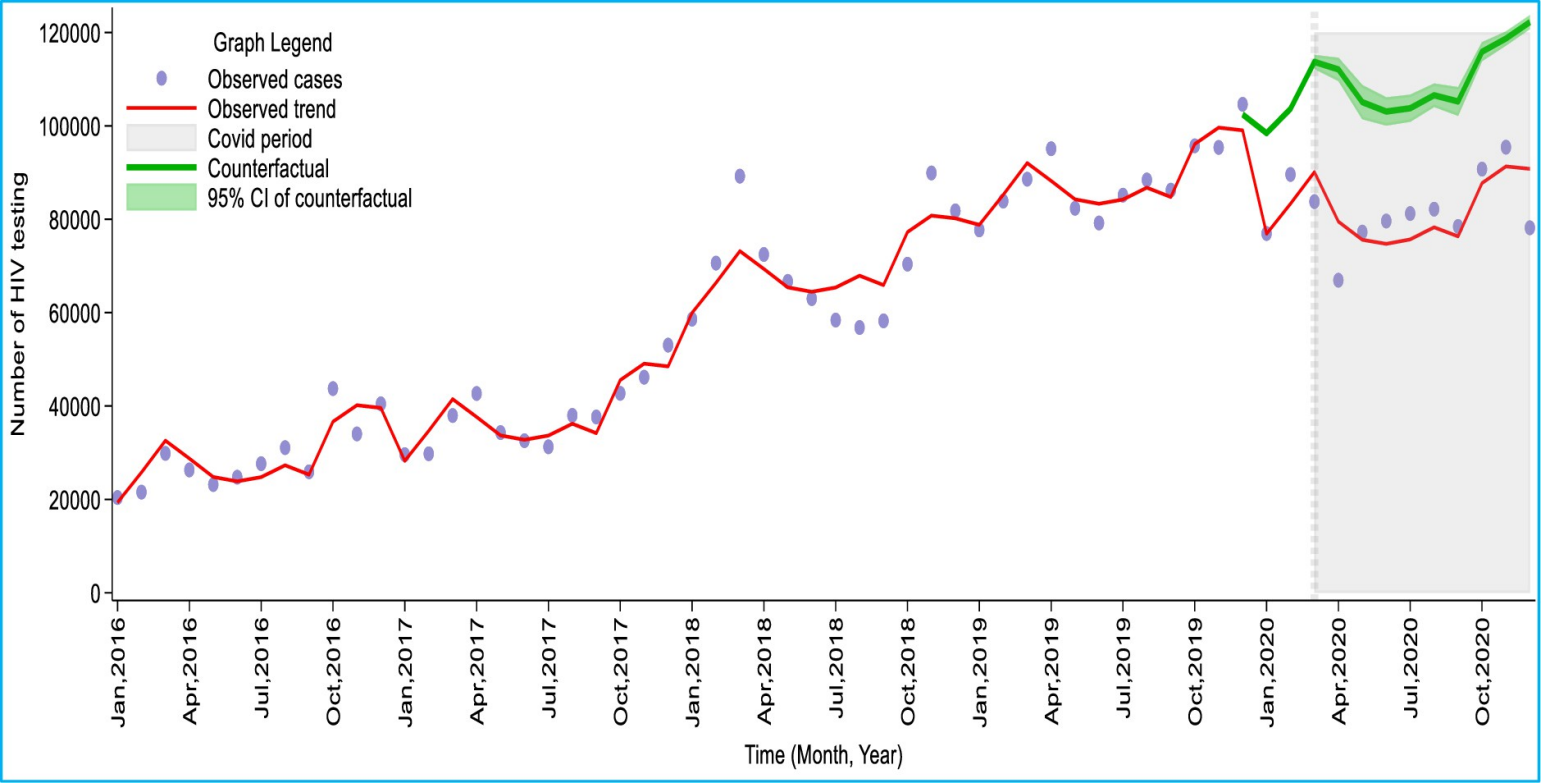
Trends of observed and counterfactuals number of HIV testing in Ghana from January 2016 to December 2020.

The regression analysis showed a 40.3% (95% CI: 38.9%, 42.6%) reduction in HIV testing done in April 2020 in Ghana compared to the counterfactual scenario of no COVID-19. After April 2020, HIV testing decreased by an average of 20% per month from May to November 2020. The trend however returned to nearly the April 2020 level, reaching an estimated 44,047 (36%) reduction in HIV tests done in December 2020. Overall, an estimated median of 22.9% (95%CI: 21%, 25%) of HIV tests were lost per month during the COVID-19 period. A cumulative loss of 262,620 or 26.5% (95%IC: 24.7%, 28.1%) of HIV tests was observed nationwide by the end of December 2020 owing to the COVID-19 pandemic (Table 2 and Fig 2).

**Table 2:** Modelled effect of the COVID-19 pandemic on HIV testing and counselling (HTC) in Ghana, from April to December 2020.

Month	Observed No. of HIV testing (Pandemic)	Counterfactual No. of HIV testing (No pandemic) [95% CI]	Difference	
			Absolute [95% CI]	Relative (%) [95% CI]
April	66,945	112,100 [109624, 114576]	45,155 [42678, 47631]	40.3 [38.9, 41.6]
May	77,254	105,086 [101435, 108737]	27,832 [24181, 31483]	26.5 [23.8, 29.0]
June	79,613	103,074 [100059, 106089]	23,461 [20446, 26476]	22.8 [20.4, 25.0]
July	81,219	103,767 [100877, 106657]	22,548 [19658, 25438]	21.7 [19.5, 23.9]
August	82,163	106,552 [104046, 109058]	24,389 [21883, 26895]	22.9 [21.0, 24.7]
September	78,454	105,224 [102160, 108287]	26,770 [23706, 29833]	25.4 [23.2, 27.6]
October	90,731	115,878 [113860, 117896]	25,147 [23129, 27165]	21.7 [20.3, 23.0]
November	95,439	118,710 [117210, 120210]	23,271 [21771, 24771]	18.6 [18.6, 20.6]
December	78,171	122,218 [120741, 123695]	44,047 [42570, 45524]	36.0 [35.3, 36.8]
Total	729,989	992609 [970012, 1015205]	262,620 [240022,285216]	26.5 [24.7, 28.1]
Median	79,613	106,552 [104046, 109058]	25,147[21883, 26895]	22.9 [21.0, 24.7]

### Impact of COVID-19 on ART initiation

**Fig 3:**
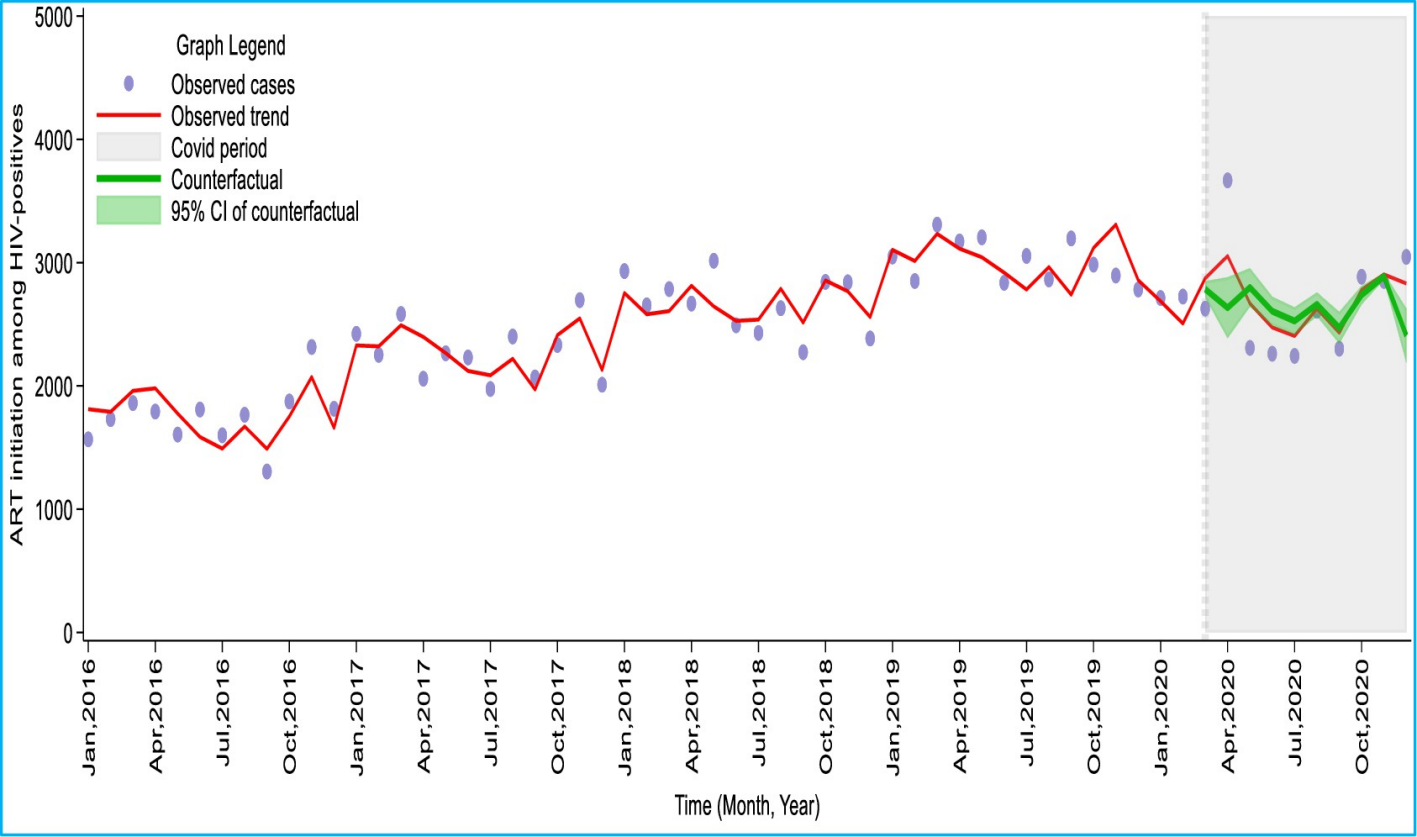
Trend of observed and counterfactual number of ART initiation from January 2016 to December 2020 in Ghana.

Table 3 and Fig 3 present the results of the time series regression analysis of ART initiations after the COVID-19 pandemic. The result showed that more (n = 3668; 39.2%) people living with HIV started ART in the first month of COVID-19 (April 2020) than what was expected under the no COVID-19 situation (n = 2635). Thereafter, ART initiations decreased by an average of about 10% per month from May to September with the highest decline in May 2020 (17.5%). The median monthly decline in ART initiations during the COVID was 2.0% (95%CI: -1.7%, 5.4%). Cumulatively, 443(1.9%) more ART initiations were done during the pandemic period (April to December 2020) than what was expected under the counterfactual scenario of no COVID-19.

**Table 3:** Modelled effect of the COVID-19 pandemic on ART initiations in Ghana, from April to December 2020.

Month	Observed No. of ART initiations (Pandemic)	Counterfactual No. of ART initiations (No pandemic) [95% CI]	Difference	
			Absolute [95% CI]	Percentage [95% CI]
April	3668	2635 [2388, 2881]	-1033 [-1280, -787]	-39.2 [-53.6, -27.3]
May	2309	2799 [2644, 2954]	490 [335, 645]	17.5 [12.7, 21.8]
June	2261	2606 [2490, 2723]	345 [229, 462]	13.3 [9.2, 17.0]
July	2244	2527 [2417, 2637]	283 [173, 393]	11.2 [7.2, 14.9]
August	2610	2662 [2567, 2758]	52 [-43, 148]	2.0 [-1.7, 5.4]
September	2302	2472 [2344, 2599]	170 [42, 297]	6.9 [1.8, 11.4]
October	2886	2737 [2663, 2811]	-149 [-223, -75]	-5.5 [-8.4, -2.7]
November	2852	2889 [2862, 2916]	37 [10, 64]	1.3 [0.4, 2.2]
December	3047	2409 [2191, 2627]	-638 [-856, -420]	-26.5 [-39.0, -16.0]
Total	24179	23736 [22566, 24906]	-443 [-1613, 727]	-1.9 [-7.1, 2.9]
Median	2610	2635 [2490, 2758]	52 [-43, 148]	2.0 [-1.7, 5.4]

## Discussion

### Impact on TB case notification

This study estimated that the pandemic caused a substantial immediate decline in the number of TB cases detected and notified during the early periods of the COVID-19 pandemic, with an average reduction of about 27% per month in the first six months compared to the same period in the previous 4 years. TB case notifications, however, increased and reached near pre-COVID-19 levels within 8 months of the pandemic. This study also demonstrated that there were an overall 2,128 fewer cases of TB notified in Ghana from April to December 2020 than what would have been expected in the absence of COVID-19.

In keeping with the findings of this study, several descriptive studies from Africa reported significant reductions in TB cases detected and notified during the pandemic period. In Lilongwe, Malawi, for instance, 19% fewer cases of TB were detected and reported in the COVID-19 period compared to the pre-COVID-19 period [10]. Studies from Zimbabwe [26], Nigeria [27], and Kenya [28] also reported reductions in the number of TB cases notified during the pandemic period, ranging from 24% to 33.7%. Soko *et al. [29]* reported in their time-series analysis study in Blantyre, Malawi that the COVID-19 pandemic caused a considerable decrease in TB case notification during the early periods of the pandemic, which however increased and reached pre-pandemic levels within nine months, with an overall reduction of about 24% during the period of April-December 2020.

These findings are similar to those described in previously published studies from non-African settings. In South Korea, for instance, a structural time-series analysis found a 24% reduction in TB case notifications per week after COVID-19 began [13]. In China [30], Brazil [31], and India [32], 48%, 48%, and 56% reductions in TB case notifications were reported, respectively, in the early months of the pandemic compared to previous years without COVID-19. McQuaid and colleagues observed in their study to assess the inequalities in the COVID-19 impact in high TB burden countries that about 38% of children and 21.8% and 28.5% of adults and the elderly, respectively, missed or delayed TB diagnosis in 2020 due to COVID-19 disruptions with no evidence of difference by age or sex at the global level [33]. In 215 countries across the world, Ranasingh et al. observed a notable decline in TB notifications in 2020 compared to the previous year in all children age groups and all regions except the African region [34]. According to the 2021 global TB report, there was a fall of about 1.3 million incident cases of TB reported globally in 2020, equivalent to an 18% drop between 2019 and 2020, with countries such as India, Indonesia, the Philippines, and China being the largest contributors of the global shortfall [6].

The variations in the trends of TB case notification among countries during the COVID-19 period and the reduced number of cases notified in the initial months may be due to differences in the severity of the impact of the pandemic, the degree of restrictions placed on the population and the extent to which they were adhered to, and the capacity and resilience of the country’s health systems [6]. During the early periods of the pandemic, governments implemented restrictive measures, often termed ’lockdowns’, including either stay-at-home orders or restrictions on movements in the population, aimed at reducing close-proximity contacts to contain the virus from spreading [35]. While some countries, such as India [36] experienced total lockdown for several months, others such as China [37] and Ghana [38] locked down only parts of the country for only a few weeks to a few months. Others such as South Korea did not order national or regional lockdowns throughout the pandemic period [35].

The overall reductions in the number of cases of TB detected and notified could be due to actual decreases in transmission associated with physical distancing and the use of nose masks. According to the Stop TB Partnership, physical distancing could decrease TB transmission by 10% in high TB burden countries [39]. However, the declines reported in this study and other studies from different settings during the pandemic may suggest the contribution of many other factors. Firstly, the diversion and reprioritizing of health workers, financing, and medical supplies from TB programmes to the COVID-19 response has been reported in many countries as both diseases present with similar symptoms and require similar infrastructure, skills and expertise for their diagnosis and management [6, 12, 39]. For example, a survey by Stop TB Partnership (2020) of 20 highly burdened TB countries found that at least 40% of national TB programmes were using facilities and resources meant for TB programmes for the COVID-19 response. This, therefore, meant that health facilities had reduced capacity to provide uninterrupted services such as TB contact tracing and diagnosis, which might have caused case detection to decline [40].

Secondly, the fear among the general population of contracting COVID-19 at health facilities coupled with governmental measures such as lockdowns and the mandatory wearing of nose masks might have led to a change in health-seeking behaviour as well as reduced access to health services in general. As in many countries, the government of Ghana implemented physical distancing measures following the onset of community transmission of COVID-19 by placing a ban on all public gatherings and urging residents to stay at home [41]. The government further imposed a three-week lockdown on the country’s major cities during the early periods of the pandemic [38]. On April 25, 2020, the Ministry of Health issued a directive ordering all Ghanaian residents to wear nose masks where physical distancing is not possible and made mask-wearing mandatory for all in public places, including health facilities. All of these factors limited access to and the availability of health services in general, particularly during the early period of the pandemic.

Thirdly, stigma, associated with infectious diseases, is a well-known phenomenon in the African setting, with HIV, TB, and Leprosy being historical models. Survivors of the recent Ebola outbreak in the Western part of Africa were faced with stigmatization when they returned to their communities [42]. It is not unexpected therefore to be scared of being known as a COVID-19 patient in this part of the world. Similarities in the TB and COVID-19 clinical presentations not only can create confusion in diagnosis, but TB patients who are already stigmatized will even be more likely to be viewed with concern. Stigmatization could lead to people being reluctant to visit health facilities for care or testing when they have symptoms related to COVID-19 that may result from TB, which may lead to underreporting of cases [43].

All of these factors could cause delays in the diagnosis and initiation of anti-TB treatment. Not only can a late diagnosis of TB and treatment delays increase the risk of transmission, especially in the household as people were ordered to stay at home [44] but may also increase the risk of adverse treatment outcomes such as deaths and the development of drug-resistant TB. For instance, evidence from mathematical modelling in three high-burden TB countries (India, Kenya, and Ukraine) showed that a 3-month disruption of TB services and a prolonged 10-month return to normal could result in additional 6.3 million cases and 1.4 million deaths globally between 2020 and 2025 [45]. Hogan and colleagues estimated in their study that the COVID-19 pandemic has the potential to increase deaths due to TB by up to 20% over 5 years. This could undoubtedly cause a setback in the progress made to eliminate TB as a public health threat [46]. Low TB case detection and notification is one of the major problems facing the TB control programme in Ghana. Evidence shows that only a third of the expected cases of TB are detected and reported in the country annually [5] and this could be exacerbated by the COVID-19 pandemic if immediate mitigating measures are not taken.

### Impact on HIV testing and counselling and ART initiation

HIV testing is the first step toward initiation into the HIV continuum of care. The UNAIDS, therefore, recommends all countries achieve universal testing and treatment for HIV in an attempt to control the HIV epidemic and reach an HIV-free world by 2030 [47]. In line with this, the following bold targets were set: by 2020, 90% of all people living with HIV should be diagnosed, 90% of all HIV-positive individuals should receive ART, and 90% of all people put on ART should achieve viral load suppression. The WHO also recommends in a new policy, the "Treat-All" approach, where every person living with HIV should be treated with ART regardless of the CD4 count or HIV clinical stage. Ghana adopted these policies and targets within its 2016–2020 national strategic plans [21]. Among the HIV testing approaches being employed in Ghana are provider-initiated testing and counselling (PITC), client-initiated testing and counselling (CITC), community-based testing and counselling, assisted partner notification and index client testing, and routine antenatal testing. All these testing methods are free of charge for everyone who uses the services [48].

This study demonstrated that the COVID-19 pandemic had a considerable immediate and sustained effect on HIV testing in Ghana, by causing more than a 40% decrease in the number of people presenting for HIV testing in the first month (April) of the pandemic, and a sustained effect of 36% as of December 2020 compared to a counterfactual scenario of no COVID-19. On the other hand, the number of HIV-positive patients starting ART increased by about 39% in the first month of the pandemic but decreased by an average of 14% per month in the following three months of 2020 compared to the same period in the previous years. There was an insignificant increase of about 2% in the number of HIV-positive patients starting ART from April-December 2020, with a positive effect as of December 2020.

These findings suggest that ART services for people living with HIV were largely not disrupted. However, absorbing new people into HIV testing and counselling and subsequent treatment was hampered by the pandemic throughout 2020.

The declined HIV testing rate has the potential to cause a setback in the gains made in controlling HIV in the country and could worsen the HIV epidemic. The consequence of a late or delayed diagnosis of HIV infection is that it can result in much higher odds of increased morbidity and mortality [49]. Testing is the only way to achieve early diagnosis since HIV infection may remain symptomless for several years, allowing for early referral for a continuum of care and support, including ART initiations, resulting in improved prognosis [50, 51]. Additionally, evidence has shown that HIV diagnosis reduces the chance of engaging in risky sexual behaviours. Persons diagnosed with HIV are more likely to use condoms consistently than those who are HIV-negative, and reduced levels of unprotected sex have been reported after an HIV diagnosis [52]. An HIV diagnosis has also been demonstrated to motivate most men to considerably reduce their risky sexual behaviours [53]. Early diagnosis of HIV thus has a great public health benefit, hence increasing the HIV testing and counselling uptake rate should be a key public health priority.

Consistent with the findings of this study, descriptive studies in Kenya reported reductions of about 15 to 30% in HIV tests done in April 2020, compared to what was done per month from January to March of the same year [54, 55]. In China, an interrupted time series analysis reported a 49% reduction in HIV tests done during the first 3 months of implementing COVID-19 response measures, compared to the counterfactual scenario of no COVID [56]. In South Africa, a similar study reported that 47.6% fewer HIV tests were done at the beginning of COVID-19 (Dorward et al., 2021). On the contrary, however, the South African study found a decrease of 46% in ART initiations in the early periods of the Pandemic [57]. The reasons for the increase in ART initiations in April 2020 observed in this study cannot be directly inferred. However, A similar study in rural South Africa found a 20% increase in overall HIV service visits during the early periods of COVID-19. The study though did not differentiate between HIV testing and ART initiations, the authors postulated that the increase in the use of HIV services could reflect the haste to collect ART as they anticipate that there will be further restrictions of movement or shortages of drugs [58].

In addition to some of the aforementioned reasons including the fear of COVID-19 contagion among the general public and health workers, stigmatization, repurposing of resources, as well as restriction of movements and lockdowns, the decline in the HIV tests during the pandemic period could also be due to reduced exposure to sexual risk behaviours [59] as most people, especially those of the high-risk populations, spent the greater part of their time at home as they tried to practise social distancing, particularly during the early periods of the pandemic to avoid exposure to COVID-19. This reason is possible as a study in China showed that more than two-fifth of the participants reported reduced sexual partners due to COVID-19 [60]. With the reduced chance of having sex, people might be optimistic about a reduction of risk of exposure and thus were less likely to go for an HIV test during the period. Studies in Uganda [61] and Kenya [55] suggest that the decline in HIV testing could be due to a shortage of PPEs, and staff being reassigned from HIV testing to COVID-19 response as well as reduced opening times of clinics. The declines in ART initiations in some months of the pandemic period could be associated with the occasional shortages of ARV drugs. Aba Abraham and colleagues for instance, reported in their qualitative study that assessed the effect of the pandemic on ART services at the Cape Coast Teaching Hospital in Ghana that in addition to the erratic client clinic attendance during various stages of the pandemic, the pandemic also caused irregular supply of some resources due to affected last mile delivery as a result of the lockdown in Accra. [62]. The shortfalls of TB and HIV services observed in this study are comparable to other health service disruptions in Ghana. Ahmed et al. for instants in their time series analysis using administrative data from 18 Low-and-Middle-Income countries, including Ghana, observed 18% and 3.7% significant reductions in general outpatient consultations and BCG vaccinations, respectively, than expected in Ghana [63]. Similarly, Amousou and colleagues reported shortfalls in outpatient (21%), delivery (3.1%) and measles vaccination (5.1%) utilizations in Ghana in 2020 due to COVID-19 [64], suggesting that the pandemic generally caused disruptions in many areas of health service utilization and delivery in Ghana.

To mitigate the effect of the pandemic on health workers and to ensure that they stay at the post to provide continual health services, the Ghanaian government, on April 5, 2020, declared its plan to hire qualified but unengaged health workers to fill staffing gaps and incentivised all public-sector health workers by giving them tax exemptions on their earnings. The government also paid an additional 50% base salary bonus to front-line health workers and promised them a life insurance cover of up to about US$60,345 per person against COVID-19 infection or death [65]. These measures increased access to and availability of routine health services including TB and HIV screening and diagnosis, and treatment, which might have minimised the effect of the pandemic in Ghana.

### Strengths and limitations

The strengths of this study are akin to those of the methods used in general. Firstly, the use of multiple time points (51 months pre-COVID and 9 months post-COVID) in this study provides a robust causal power to detect any effect of the pandemic [66]. Secondly, unlike most of the past studies from different settings that used clinic-specific data to quantify the effect of the pandemic, which limits generalization, this study used nationally representative data. Thus, the findings of this study are generalizable to the target population.

However, the study is not without limitations. To begin with, though data entered in the DHIMS2 database are systematically checked for accuracy and completeness, there was a possibility of the presence of missing data along the reporting cascade, which this study could not validate. Secondly, the study did not assess from the perspective of TB and HIV patients how the pandemic affected their health-seeking and access to healthcare. Notwithstanding these weaknesses, the study has provided important insight into how the COVID-19 pandemic affected TB and HIV indicators in its first year, useful for programme planning.

### Conclusion

This study has demonstrated that the pandemic had a substantial negative impact on aspects of TB and HIV health services, particularly during the initial periods of 2020, as reported in many previous studies from different settings. It was estimated that the pandemic caused a substantial immediate effect on the number of cases of TB notified during the period. On the other hand, the engagement of people in HIV testing was severely affected by the COVID-19 pandemic throughout 2020. ART initiation was generally not affected. Aggressive case-finding campaigns to actively find the thousands of individuals with TB that were missed over 2020. Campaigns can take various forms including, facility-based, or community-based campaigns, depending on resource availability, to promote and increase TB screening and diagnosis. In addition to continuing facility-based testing, District health services should promote differentiated HIV testing schemes, with emphasis on scaling up of self-testing approach, particularly for populations not being reached by facility testing. Community HIV testing, including, for example, at pharmacies, or Community-Based Organisation (CBO) testing services should be prioritised. Community-based ARV distribution and ART initiation for people with HIV could be a viable alternative when deemed safe bearing in mind the COVID-19 repercussions on the community.

## Supporting information

S1 Dataset Dataset analysed for this study. (XLSX)

S1 Appendix Trends of observed and counterfactual number of TB case notifications, HIV testing and ART initiations, 2016–2020. (DOCX)
