# Supplementary material for: Impact of COVID-19 pandemic on Tuberculosis and HIV services in Ghana: An interrupted time series analysis
Source: PLoS One. 2023 Sep 20;18(9):e0291808. doi: 10.1371/journal.pone.0291808 (PMC10511085; doi:10.1371/journal.pone.0291808)
Supplement: Supplementary file 1 [file pone.0291808.s002.docx]

A

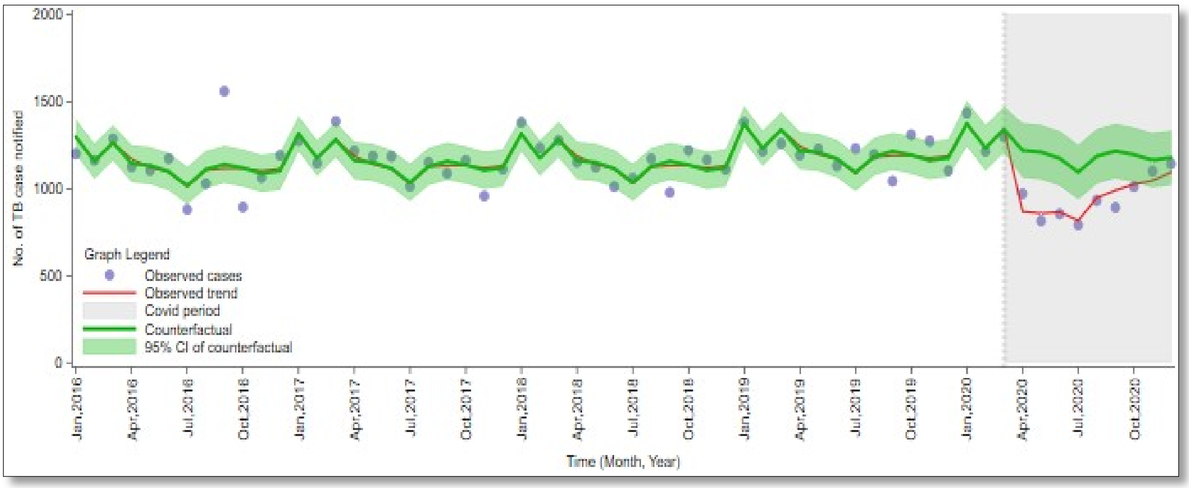

B

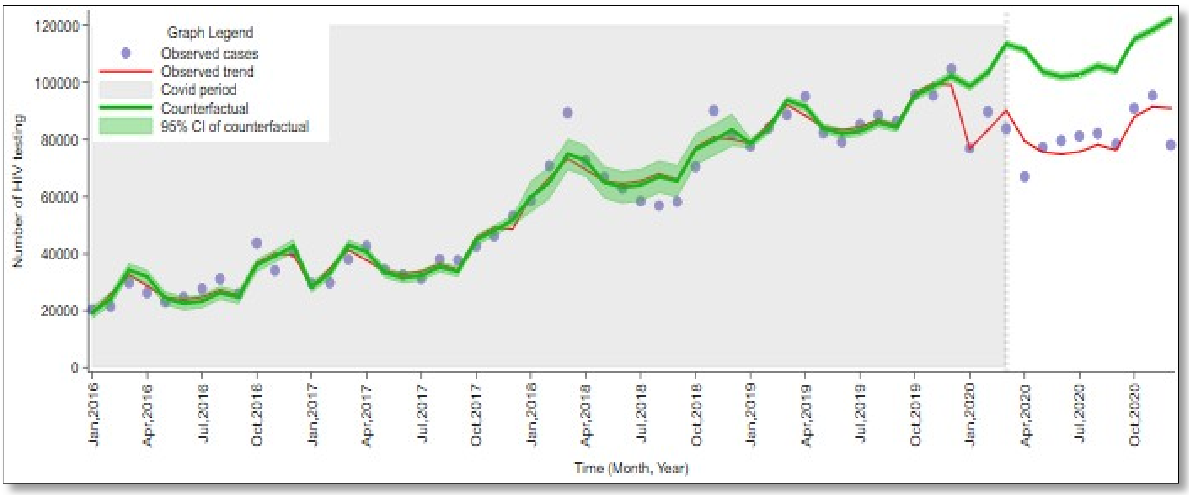

C

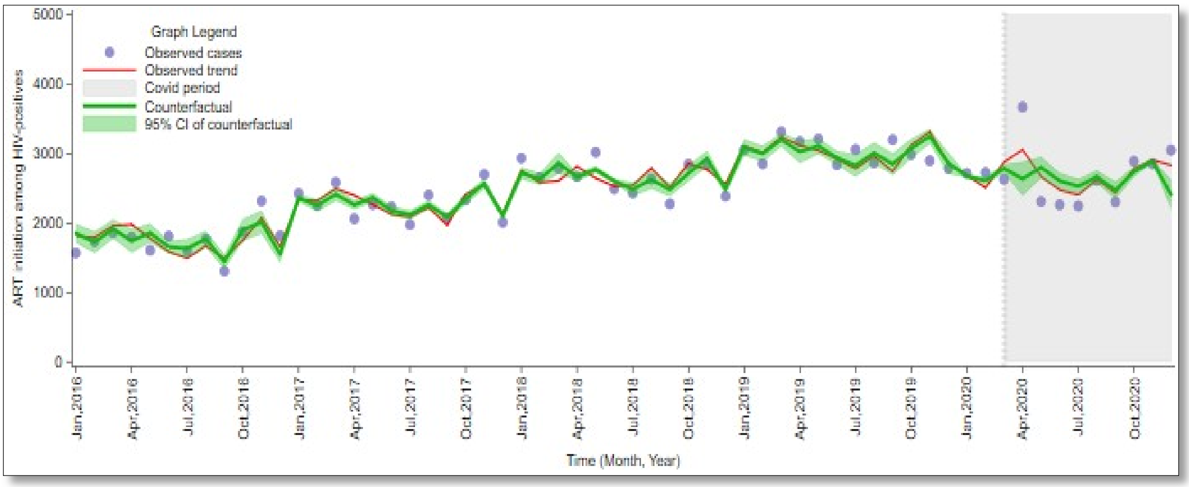

Trend and counterfactual of the number of TB case notification (**A**), HIV testing (**B**) and ART initiation (**C**) from January 2016 to December 2020 in Ghana
